# Medical and Physical Intelligence

**Published:** 1825-09

**Authors:** 


### MEDICAL AND PHYSICAL INTELLIGENCE.

### PHYSIOLOGY.

1. *Structure of the Nerves.*—M. Bogros read a Memoir relative to the Structure of the Nerves, before the Royal Academy of Sciences. In this memoir the author observes, that two substances enter into the composition of the nerves,—the *neurilema,* composed of cellular tissue, and the *medullary fibre.* The ganglia were considered as composed of two parts: the medullary threads, in fact, penetrate into them, are stripped of their neurilema, are rolled up, and (as it were) united together by a particular substance, sometimes greyish, at others yellow or reddish.

M. Bogros subjected the nerves to new experiments; from which it results, that, independently of the neurilema and the pulp, a central canal is to be recognised. With the assistance of tubes, nearly resembling those by which mercury is injected into the lymphatic vessels, but with finer points, he succeeded in injecting the nerves. No preliminary preparation is required for the experiment; it was even performed upon living animals. The following are the principal facts related:—

When a nerve is pierced by the point of a prepared tube filled with mercury, the injection runs through all the filaments furnished by the nervous chord, to their furthest extremities: it may be traced in the papillæ of the skin, the mucous membranes, and muscles; the injection also extends itself towards the origin of the nerve; and, lastly, when thrown into a single filament, it always extends to several others, by means of anastomosing canals. If, after having injected the nerve, it is cut, a round and regular opening is to be observed in the centre of the pulp; and, even without the injection, if the centre of the pulp is carefully examined, an obscure point may be seen,—that is the opening above mentioned; and, by placing the point of the tube in this place, the nerve is injected.

When a nerve is deprived of its neurilema by nitric acid, similar results are obtained: when, on the contrary, the pulp is destroyed by an alkaline ley, injection is performed badly; it stops, and does not present the same regular and cylindrical aspect. Lastly, if spirits of turpentine are injected, and the nerve is then dried, the structure of the canals is visible to the eye. Mercury flows through the filaments of the great sympathetic nerve, and shows canals similar to those found in the other nerves. Thus, an injection thrown into the inferior cervical ganglion has penetrated through the cardiac nerves to the heart; and, from the great sympathetic, it has reached the semilunar ganglion, and the branches that extend from it. When the injection reaches the ganglia, they swell, and present the appearance of a number of canals communicating with each other, and turned and twisted upon themselves. The injection of the intervertebral ganglia takes place in a peculiar manner: they swell immediately; afterwards the injection penetrates into the venous network, situated between their proper surface and the covering with which they are furnished by the dura mater, and thence into the veins of this membrane itself. At last, the injection is seen to pass across the roots, and to fall into the cavity of the dura mater; a result which arises either from ruptures easily made in this point, where the pulp is very soft, or from this effusion taking place through natural openings. An injection cannot be thrown into the roots, nor *a fortiori* into the spinal marrow: it could not pass above half an inch when the pulp was torn, from whence resulted an opening through which the mercury escaped. The injection penetrates into the veins sometimes, but never into the arteries or lymphatics. The anastomoses take place by the meeting of the medullary canals, or by a confusion of the pulps: where they take place, the nerve increases in size.

The injection has been performed on living frogs. At the beginning, convulsions were produced; when it was finished, a perfect paralysis was produced. These experiments have been repeated on the four classes of vertebrated animals.

In an anatomical point of view, this discovery is very valuable; physiologically, the existence of a canal in the nerves opens a vast field of inquiry; and as there is a law in the animal economy, that every cavity which is not lined by a mucous or serous membrane, is closed by an adhesion of its sides, if it is not preserved by the presence of some other body, is it not, therefore, probable that the medullary canal serves for a kind of circulation?—(*Archives Generales*, Juin.)

### PATHOLOGY.

2. *Question as to the Contagion of Yellow Fever.*—Dr. Cherwin, in order to settle the above question, has employed himself in the most painful and dangerous researches; the whole conducted entirely at his own expense. He left Paris in October, 1814, and arrived at Guadaloupe in December of the same year: he has since visited Cayenne, the Antilles, the coasts of North America (from Louisiana to the main), and the whole of Spanish South America, with the purpose of witnessing this fever,—collecting information from every possible source,—and examining all the public and private documents bearing upon the question. He, in the years 1816 and 1817, opened the bodies of upwards of five hundred persons who had died of this disease; besides many others during the terrible epidemics of New Orleans, and especially that of the Savannah in 1820. The result of these immense labours is, his firm conviction that yellow fever is produced only by local causes, put into action by a peculiar constitution of the air, which it is, however, difficult to appreciate; that the local cause consisted of emanations from vegetable or animal substances in a state of putrefaction; and that the disease was never propagated by contagion, in any of those instances which came to his knowledge.—(*Bulletin des Sci. Med.* Juin.)

3. *On the Mean Duration of Intermittent Fever.*—M. Bailly has read, before the Royal Academy of Sciences, a memoir on this subject, the result of a great number of observations collected in different climates,—such as Rome, Montpellier, Lyons, and Canada; and he gives a period of fourteen days as the mean duration of intermittent fever. The remarkable circumstance to be observed is, that this period is precisely that in which acute diseases run their course. This analogy offers another powerful motive to assimilate these two kinds of morbid affections; the identity of which is further proved by the traces of inflammation which are almost always found in the internal organs, as the consequence of intermittent fevers.

M. Bailly afterwards considers the physiological cause which determines a disease to prolong its course for a definite period of time. Inflammation, according to him, is not merely the result of an accumulation of blood in this or that organ; it consists in a fixed and permanent alteration of the diseased tissue, and this alteration can only be destroyed by those changes which nutrition determines; but, as these changes are necessarily slow and successive, it follows that every inflammation must occupy a fixed time to arrive at its maximum, and disappear. Experience can only determine how many organic changes are necessary to destroy that alteration in a tissue which constitutes a state of inflammation; and, if intermittent fevers take two weeks to be cured, it may be concluded that the internal organs, when inflamed, require that space of time to pass through those stages necessary to reproduce a healthy state. As to the singular tendency which the greater part of diseases have to run their course by weeks, there is nothing that need surprise us; since the organic movements of the healthy state present a similar course. The first dentition generally begins at the seventh month, the second at the seventh year. Menstruation is equally regular with regard to its septenary periods.—(*Archives Generales*, Juin.)

4. *Cancer of the Uterus.*—M. BAUDELOCQUE, the nephew, relates the case of a cancer of the uterus, which he cured by the repeated application of leeches to the neck of that organ; the use of a compress covered with an opiate cerate; cauterisation, with the lunar caustic, of the fungous growth, at several successive periods; and the employment of injections, at the commencement of the disease, composed of sulphate of zinc, acetate of copper, and deuto-chloride of mercury; and, finally, during the whole course of treatment, which lasted three months, by the use of hip-baths and narcotic enemata.—(*Archives Generales*, Juin.)

5. *Cure of a Hydrophobic Patient.*—Professor ROSSI has published a very interesting case, of which we here give the outlines; premising that, from the documents annexed to the Memoir, it would appear that every proper means have been employed to prove its truth and authenticity. A person, aged thirty-two, of a sanguine temperament, in excellent health, and of great liveliness, was attacked by a strange cat on the 22d of October, 1824, which fastened with its teeth on his left hand, until with the other, which received numerous scratches, he was at length enabled to drive it away. There were three wounds on the left wrist, two of which, from the teeth of the animal, had penetrated through the skin, and bled profusely; the other was a lacerated wound, about two fingers'-breadth in length, on the other side of the hand: the parts were merely washed with cold water. The cat belonged to a neighbour, and for two days had attempted to bite those who gave it food: it was killed on the following day, having all the symptoms of rabies. At the end of twenty-four hours, a surgeon cauterised two of the wounds, but very superficially; omitting, however, the third wound, which was the most extensive. Some days after, the patient called on Dr. CASTAGNO, of Lanzo, who sent him with a letter to Professor Rossi, for his advice upon the case; in consequence of which, on the 19th of November, (that is, twenty-seven days after the accident,) the wounds were thoroughly cauterised again, and the sublingual glands were examined, in order that their subsequent condition might be watched. The patient was directed to take vinegar and the genista luteo-tinctoria, internally; of which he drank a glassful night and morning.

No alteration was perceived, either in the habit, manner, or health of the patient, until the beginning of December, when he began to be melancholy, to seek for solitude, and wept violently; his sleep was disturbed by frightful dreams, his appetite failed, and he disliked his wine, of which he had been before very fond;* his eyes glistened, his face was red, and his mouth was always full of saliva. The left sub-

* He afterwards said, that his aversion to water was at that time equally strong.

lingual gland was larger than the right, which continued in its natural condition; and he felt an itching in the part bitten.

On the 10th of December, the sublingual glands were cauterised, by the application of a heated iron button-cautery, three successive times, applied to each gland. The operation was very painful; three hours afterwards a smart attack of fever took place: rest, and a rigid regimen, were prescribed. At the end of six or seven hours, a grain of opium was given, by which he obtained three or four hours' sleep. The next day the fever, pain, and salivation, were diminished. The same means were employed, and the following day he was quite free from fever. Three or four days afterwards, he assumed his usual mode of life; and now enjoys the most perfect state of health.—(*Annali Universali*, June.)

6. *Existence of the Lyssæ in a Rabid Dog.*—A middling-sized dog, two years old, was bitten by a strange dog, in the early part of April, 1824. On the 10th of that month, he became sullen, restless, less sensible than usual to caresses; but still he continued both to eat and drink. His master took no particular care of him. On the 13th of June, this dog bit several dogs, and a little girl, with such fury, that some alarm was excited; on which account he was brought, on the following day, to the veterinary school. The general appearance of the animal did not denote any disease; but he was soon seized with so violent a paroxysm, that he fell upon every thing in his way, refused to eat or to drink, though he did not show any horror of liquids. The principal symptoms were redness of the inside of the throat, hoarse noise in barking, immobility of the jaw, fierce eyes, pupil very much dilated, and the conjunctiva inflamed; a strong light appeared to cause him uneasiness, and to bring on the paroxysms of fury. The next day the same symptoms persisted, though in a less violent degree, on account of his increased weakness. The dog died.

On opening the body, the mouth was found nearly free from saliva. On the left side of the frænum of the tongue, there was a small swelling of a longish shape, pointed anteriorly, and posteriorly extending to a level with the first molar tooth; the parietes of this swelling, dry and injected, were swollen in the middle, so as to give it the appearance of a barley-corn; the posterior extremity had, towards its termination, a small aperture, analogous to the opening of a dilated follicle. This vesicle contained a little yellowish liquid, of a tolerable consistence: within it two ulcerated spots were observed, covered with a matter the colour of milk. There was nothing particular to be seen on the upper part of the tongue; but, towards its base, a redness extended into the fauces. The stomach was full of hair, fragments of rope, and other foreign substances; the mucous surface was inflamed; and, towards the curvatures, there were some ecchymosed spots and ulcers. There were no other diseased appearances, either in the thorax or abdomen. The substance of the brain was a little softened, and rather more vascular than ordinary; and this vascularity was still more remarkable in the spinal marrow, as well as in its envelopes. These circumstances are related by M. Anthonio Soares, of the Veterinary School.—(*Ibid.*)

7. *Cases of Palsy, not in accordance with the Appearances after Death.*—M. NACQUART read a report on a Memoir of M. VELPEAU, relative to four cases of palsy. In one case, the autopsic examination discovered a great density of the whole encephalon; the conversion into pus of the whole grey central matter of the cervical spinal marrow, on the right side; a softening of the same part on the left side: nevertheless the patient, a female, had not evinced any nervous symptom during life, excepting a hemiplegia of the left side of the body, which only took place five days before her death.

In the second case, the patient had gradually lost the motion and sensibility of all the parts; and the body presented no unusual appearance, excepting a cartilaginous hardness of the cervical portion of the spinal marrow.

In the third case, the patient died of an old palsy, which had affected every part of the body in succession, excepting the thumb and index-finger of the right hand; and yet only three or four ounces of serosity were found in the ventricles of the brain, and a light grey spot, not very thick, and of small extent, in the centre of the tuber annulare, with some softening of that part.

Finally, the last patient died suddenly, whilst suffering from a vast abscess behind the sacro-iliac symphysis, and whose cerebro-spinal system was, notwithstanding, quite healthy, excepting that the sanies of the abscess had penetrated through the sacral foramina into the spinal canal, and had tinted of a brown colour the spinal arachnoid and the origin of the spinal nerves.

M. Velpeau observes, that, in these cases, there is no relation between the symptoms during life and the alterations perceived after death.—(*Archives Generales,* Juin.)

8. *Ossification of the Spleen.*—The following case tends to confirm the proofs which already exist, that, whatever office the spleen may perform in the animal economy, it is not one of primary importance to life, or even to health. In opening the body of a person who was drowned, the spleen was found converted into a hard bony substance. The periosteum (peritoneum) being taken off, this bone was found to be white and smooth; the vasa brevia were not ossified; internally, it was cellular and spongy, and contained in the middle a fleshy mass, the remains of the viscus. The most remarkable circumstance is, that the individual had always enjoyed the best state of health.—(RUST'S *Magazin fur die Gesanmte Heilkunde.)*

9. *Diagnostic of Venereal Gonorrhœa.*—The Baron VON WEDEKIND affirms, that the true diagnostic sign of the above disease being really the produce of impure connexion, is the appearance of two small lenticular knots, situated one on each side of the frœnum, immediately behind the orifice of the urethra; which little swellings may always be distinguished by the touch, and often by the eye. The Baron believes them to be small glands, and thinks that from them the contagion of gonorrhœa proceeds.—*(Ibid.)*

### THERAPEUTICS.

10. *Salivation from a Dose of Calomel.*—Mr. THOMAS, of Pembroke Dock, has transmitted to us an account of a lady, for whom he prescribed a dose of four grains of calomel, with six of jalap, which was taken on the 10th of January at night, and followed by a cathartic draught the next morning. It operated the next day as a purgative; but complete salivation was established on the 12th. Our correspondent observes, that this is the second time a similar effect had been produced by small doses of mercury in this individual. A surgeon at Plymouth, wishing to affect the mouth slightly, sent her some pills, two of which were to be taken every six hours; but, before the dose could be repeated, she was salivated.—(*Private letter from* Mr. THOMAS.)

These occurrences are not infrequent; but they serve to point out the necessity of inquiring into the peculiarities of constitution of patients, when we first prescribe for them.

11. *Oil of the Euphorbia Lathyris.*—M. CAVENTOU presented to the Royal Academy of Medicine some details relative to this oil, which is a purgative nearly as drastic as the croton tiglium. Six or eight drops are a sufficient dose; and, as it is inodorous, and nearly without taste, it may be given to infants in all sorts of vehicles, in lozenges, or in pills. It is also very reasonable in its price; one ounce, costing only a franc, will purge ninety-six persons. When it is old or rancid, it causes colicky pains. Dr. CALDERINI has used it as a suppository with the butter of cacoa.—(*Revue Medicale*, Juin.)

12. *Virtue of the Black Pepper as a Febrifuge.*—Dr. LOUIS FRANK having invited the profession to try the effect of the extract of black pepper in intermittent fever, prepared by digesting, in a glass vessel, one ounce of the pepper in twelve ounces of distilled water, for thirty-six hours, in a water bath, and evaporating the liquor to the consistence of an extract. This remedy was tried on nine individuals, affected with intermittent fever of different types, in doses of four, eight, ten, or twelve grains, dissolved in water, in some cases, given in the form of pills in others, by Dr. CLOCK, of Trent; and the effects surpassed his warmest expectations.

From these experiments, the Doctor concludes that the extract of pepper is not only one of the best succedaneums for the bark, but that it actually is very preferable to it, on several accounts: 1st. It never produces disturbance of the stomach or bowels. 2d. Because it never failed in producing its curative effect. 3d. Those who were cured did not, in any one instance, experience a relapse. 4th. It promotes a regular alvine discharge, as well as the excretions of urine and sweat. 5th. None of those who were cured experienced that sensation of languor so common to a state of convalescence.—(*Giornale de Chirurgia Practica*, March.)

13. *Sulphate of Quinine.*—M. HENRY, sen. read a paper on the mutual action of this salt and different wines. On mixing four grains of sulphate of quinine with four ounces of wine, immediately a portion of this salt is precipitated, either by the colouring matter of the wine, —by the astringent principle contained in it,—or by the tartar; a portion of the quinine remains in the wine, in the state of an acid sulphate. —(*Revue Medicale,* Juin.)

14. *Effects of the Chloruret of Lime.*—M. VIREY communicated to the Royal Academy of Medicine a table of the diseases which afflicted the army in Spain in 1812, drawn up by Dr. ETIENNE; from which it appears, that the chloruret of lime, sprinkled between the beds of patients labouring under typhous fever, produced the most beneficial effects. At the next sitting of the Academy, M. LABARRAQUE said that the mode in which the chloruret was employed on the above occasion, has no resemblance to the manner in which he uses the same substance, which is applied by him immediately to infected matters, so as to destroy putrefaction at once.—(*Ibid.*)

15. *Use of the Red Sulphate of Iron.*—M. BRACCONOT, of Nancy, has discovered that this salt (the persulphate of iron) possesses an astringent and antiseptic property in the highest degree. It is very cheap, and combines, with the greatest facility, with all the humors and soft tissues of animals, and preserves them both from putrefaction and from insects. A brain, which had been plunged for three months in a solution of this salt, being placed in a warm place, required a considerable time to dry it, but without showing the least sign of putridity: placed afterwards in water, it was still preserved for some time, but did not recover its pristine softness. Portions of the liver, spleen, lungs, and muscle, placed in this mixture, have equally resisted destruction.

The preparation of this salt is very easy: it consists in calcining the green sulphate of iron until it acquires a red colour.—(*Archives Generales,* Juin.

---

### SURGERY.

16. *Fracture and Consolidation of the Clavicle of a Fœtus in Utero.* —M. DEVERGIE reported the case of a woman, who, in the sixth month of her pregnancy, struck her abdomen violently against the edge of a table, in falling from an elevated chair. The pain was very severe, and lasted a considerable time: at length, however, it disappeared, and at the usual time she was brought to-bed of a healthy child, but which presented a large tumor in the region of the left clavicle. The child died on the eighth day; and, on examining the body, a fracture of the clavicle was discovered: the two fragments of the bone had wrapped over each other a little, and were united by a solid and voluminous callus, which formed the above-mentioned tumor. The two ends of the bone had also acquired a growth much greater than is met with in a healthy state. The author attributes this accident to the fall which the mother received two or three months previously.—(*Bulletin des Sciences Med.* Juin.)

17. *Cure of Cataract and Gutta Serena.*—Dr. GONDRET reports, that the cauterisation of the frontal portion of the cranium, either by means of heated copper or by ammonia, cures, as well as prevents, both cataract and gutta serena. The Doctor says that, by the use of these means, together with the continued employment of galvanism, he has succeeded in curing many aged persons of these affections, and has improved the condition of many others.—(*Revue Med.* Juin.)

18. *Calculi retained in the Urethra.*—Dr. BOULU read the case of a child, in whose urethra a calculus had been stopped, just anterior to the scrotum, causing a retention of urine, which was extracted through an incision made in the lower part of the penis. M. AUMONT said, that he had several times succeeded in obtaining the expulsion of similar calculi, by passing into the urethra, down to the spot where the calculus was fixed, a gum-elastic bougie, and suffering it to remain there. By this means the passage became dilated on this side of the foreign body, which is thus forced out by the impulsion of the urine. M. BAFFES reported a case still more curious: it is that of a child, in whose urethra a stone was also impacted; the father of the child succeeded in disengaging and expelling it, by means of suction performed with his mouth. —(*Archives Generales*, Juin.)

### MIDWIFERY.

19. *Extra-Uterine Fœtus.*—M. BAUDELOCQUE read a report upon the case of extra-uterine pregnancy, of which mention was made in a previous sitting of the Royal Academy of Medicine. The fœtus appears to have been contained in a thick coriaceous cyst, containing in its substance some calcareous concretions, and appearing to be developed in the left fallopian tube. The fœtus has all the characters of one of seven or eight months: it is rolled up in the shape of a ball, and bent forwards. The tumor which was found in the abdomen of the mother had existed for ten years, during which space of time the fœtus had remained confined in the abdominal cavity. The author of this case has not been able to obtain any precise information as to the history of this woman prior to her pregnancy, nor of the symptoms that attended its commencement.—(*Bulletin des Sciences Medicales*, Juin.)

### NATURAL HISTORY.

20. *On the Reproduction of the Officinal Leech in the Antilles.*—M. ACHARD, of Martinique, has recommended to the Society of Pharmacy at Paris an interesting notice on the above subject. This gentleman, perceiving the difficulty of keeping the leeches imported from Europe, and finding that the small native leech does not suck the blood of the human subject, was led to make trial of those means which bear the greatest analogy with the places and situations where the European medicinal leech is bred and multiplied; and, in conjunction with Dr. LEFORT, he instituted some experiments, of which the following is a summary:—He placed at the bottom of a glazed earthen jar, containing from fifty to sixty litres, a quantity of the clay of the country, of the consistence of soft paste, and about ten centimetres in thickness. Two hundred of the largest leeches, the grey and green sorts, were placed in this vessel, which was covered by a strong cloth; taking care now and then to pour on them a sufficient quantity of water to keep the clay moist. This method succeeded perfectly, so that only here and there a dead leech was found. The vases were visited every three days: sometimes two, and sometimes three, leeches were found dead on the surface of the clay; but, in the interior, no dead ones were found. It appeared that, when sick, they came up to the surface to breathe more at their ease, and generally died there.

With regard to the reproduction of these animals, M. Achard says, that, having read Mr. NOBLE's Memoir, he perceived the cocoons, as described by that author, sometimes within, sometimes on the surface of the clay, and which he threw away, conceiving them to have been the formation of some insects; having, on opening them, only found some gelatinous matter, and a little bloody water within them. However, after reading the above memoir, he visited his vases, and found three of these cocoons; and, in the presence of Dr. Lefort, they were placed in a bottle half full of water, where, in about twenty-five days, he had the satisfaction of discovering the young leeches in the shape of fine threads, the colour of flesh and transparent, with a gelatinous matter adhering to them, together with a fluid about the consistence of syrup, and having an ammoniacal smell. Two of these lived: they did not grow much, but acquired the greenish colour of their species.

M. Achard has since constructed a large wooden box, where he preserves in this way above two thousand leeches. We have not space to insert his description of the manner in which the eggs are formed.—(*Journal de Pharmacie,* Juin.)

---

### MISCELLANEOUS.

21. "*The Living Skeleton.*"—Our country readers may, perhaps, expect that we should say something respecting the "living skeleton," at present exhibiting in London. We have visited this unfortunate individual, and shall shortly mention those circumstances in his appearance which particularly attracted our attention, without any reference to what has been written elsewhere upon the subject; which, indeed, as we have not read, cannot possibly have biassed our judgment.

This poor man is, we should conceive, about five feet seven inches in height, and is stated to be twenty-eight years of age; yet, notwithstanding the projection of the cheek-bones, the falling-in of the cheeks, and the peculiarly pale, or rather livid, complexion of the countenance, he certainly does not appear older,—scarcely, we think, so old. His general appearance gave us the idea of a person in the last stage of some chronic disease: his extreme emaciation, the expression of his countenance, the languor and want of energy in his movements, all contribute to produce this impression. Upon a more leisurely survey, the attention is, however, particularly directed to the formation and condition of the trunk and upper extremities; for neither the thighs nor the legs, more especially, are so much wasted as we have frequently seen them:

indeed, the legs, particularly the right, are by no means deficient in muscle; the gastrocnemii are sufficiently marked; whilst, in the thigh, the course of the sartorius muscle is distinctly traceable.

The arms are not the least remarkable traits in the appearance of this extraordinary individual. The fore arm certainly shows something like shape and muscle, though in a very faint degree; but the upper arm scarcely exhibits a trace of any thing but bone covered with integument, and it is curious to observe how the limb is moved and raised in almost every direction, to a position nearly horizontal, apparently without the existence of muscle.

The trunk of the body presents several deviations from a natural formation. The sternum, instead of being externally convex or flat, is bent inwards, so as to contract the dimensions of the thoracic cavity very much, especially at its upper part; the space between the sternum and vertebral column being extremely limited indeed. Lower down, the ribs form a larger curve than usual; and, in consequence of the spine being somewhat inclined to the right side, the left appears considerably more full than the right side of the thorax, and the pulsations of the heart are felt between the seventh and eighth ribs. The thoracic viscera appear as if they were forced below their usual situation, in consequence of the contracted space of the upper part of the chest, especially as far as regards its depth. The abdomen occupies a very small space, and sinks inwards towards the spine; the distance between the edge of the lower ribs and the cristæ of the ilium, is also remarkably small.

The position of the scapulæ is likewise very peculiar: instead of lying flat against the posterior surfaces of the ribs, the inferior costæ project considerably outwards, in consequence of the superior costæ being thrown so far forwards as nearly to reach the clavicle; so that the muscles arising from the cervical vertebræ, and inserted into this part of the scapulæ, being shorter and broader than natural, together with the projecting bone, cause the individual to appear both short-necked and high shouldered; which, together with the peculiar colour of the skin, and more especially of the countenance, induce the belief that respiration is performed laboriously, and that the blood is imperfectly decarbonised.

The skin felt cold and dry to the touch; the pulse was small, and beating about sixty strokes in the minute; and the surface of the body seemed to be quite destitute of hair; the voice was by no means remarkably deficient in strength.

Such are the principal circumstances, which we can vouch for from ocular demonstration. In addition, we are informed that the man eats and drinks very sparingly, but that he digests and sleeps well; that his excretions are regular and natural; his mental faculties are sufficiently developed; and, finally, that the sexual passion also exists, though it has never been indulged.

### METEOROLOGICAL JOURNAL,

**By Messrs. William Harris and Co. 50, Holborn, London.**

***From July 20 to August 19, 1825.***

| | Moon. | Rain gauge | Therm. | | | Barom. | | De Luc's Hyg. | | Wind. | | Atmospheric Variations. | | |
|---|---|---|---|---|---|---|---|---|---|---|---|---|---|---|
| | | | 9 A.M. | Max. | Min. | 9 A.M. | 10 P.M. | 9 A.M. | 10 P.M. | 9 A.M. | 10 P.M. | 9 A.M. | 2 P.M. | 10 P.M. |
| July | | | | | | | | | | | | | | |
| 20 | | | 73 | 79 | 58 | 30.17 | 30.16 | 64 | 64 | E | SE | Fine | Fine | Fine |
| 21 | | | 64 | 72 | 51 | 30.14 | 30.11 | 67 | 64 | NE | ENE | Cloud. | | |
| 22 | ☽ | | 62 | 70 | 55 | 30.10 | 29.88 | 62 | 55 | NE | W | Fine | | |
| 23 | | | 61 | 67 | 50 | 29.93 | 29.93 | 64 | 59 | ENE | NE | Cloud. | Cloud. | |
| 24 | | | 60 | 67 | 50 | 29.97 | 29.08 | 61 | 67 | NE | NE | Fine | | |
| 25 | | | 60 | 68 | 55 | 30.17 | 30.17 | 62 | 55 | NE | NNE | Fine | | |
| 26 | | | 62 | 68 | 52 | 30.18 | 30.17 | 66 | 64 | NNE | NNE | Cloud. | | |
| 27 | | | 62 | 70 | 54 | 30.13 | 30.13 | 66 | 74 | NE | ESE | Fine | Fine | |
| 28 | | | 66 | 78 | 55 | 30.09 | 30.09 | 61 | 69 | ENE | ENE | Fine | | |
| 29 | ○ | | 64 | 73 | 50 | 30.08 | 30.05 | 64 | 66 | E | E | | | |
| 30 | | | 63 | 73 | 56 | 30.04 | 29.93 | 66 | 52 | E | SE | | | |
| 31 | | | 68 | 80 | 60 | 29.93 | 29.90 | 65 | 53 | E | SE | | | |
| Aug | | | | | | | | | | | | | | |
| 1 | | | 66 | 81 | 67 | 29.90 | 29.90 | 66 | 53 | SE | W | Cloud. | | |
| 2 | | | 72 | 77 | 59 | 29.88 | 29.88 | 56 | 69 | W | SW | | Rain | Cloud. |
| 3 | | .28 | 64 | 72 | 60 | 29.85 | 29.85 | 72 | 69 | SW | SW | | Cloud. | |
| 4 | | | 69 | 71 | 57 | 29.57 | 29.23 | 76 | 79 | W | SW | | Rain | Rain |
| 5 | | .27 | 64 | 71 | 56 | 29.40 | 29.53 | 66 | 64 | NW | W | | Fine | |
| 6 | ☾ | | 60 | 75 | 55 | 29.55 | 29.58 | 72 | 69 | W | W | Rain | Rain | Fine |
| 7 | | .27 | 65 | 71 | 57 | 29.68 | 29.72 | 65 | 57 | W | W | Fine | Cloud. | Cloud. |
| 8 | | | 62 | 69 | 59 | 29.59 | 29.61 | 71 | 56 | WSW | W | Rain | Fine | Fine |
| 9 | | | 63 | 70 | 53 | 29.61 | 29.60 | 70 | 65 | W | W | Fine | | |
| 10 | | | 59 | 65 | 53 | 29.54 | 29.71 | 71 | 69 | W | NNW | Fine | Rain | Rain |
| 11 | | .25 | 59 | 66 | 56 | 29.87 | 29.95 | 74 | 52 | N | N | Cloud. | Fine | Fine |
| 12 | | | 66 | 70 | 59 | 29.96 | 29.86 | 75 | 60 | NW | W | Fine | | |
| 13 | | | 62 | 65 | 59 | 29.60 | 29.30 | 80 | 66 | WSW | W | Cloud. | Cloud. | Cloud. |
| 14 | ● | | 63 | 67 | 49 | 29.33 | 29.34 | 67 | 69 | WNW | NW | Fine | Fine | Fine |
| 15 | | .38 | 57 | 67 | 56 | 29.37 | 29.54 | 78 | 77 | WNW | NW | Rain | Cloud. | Cloud. |
| 16 | | | 61 | 68 | 59 | 29.71 | 29.75 | 79 | 72 | NNW | W | Rain | | Fine |
| 17 | | .05 | 68 | 65 | 58 | 29.75 | 29.77 | 66 | 76 | NW | WSW | Fine | | Rain |
| 18 | | | 61 | 68 | 57 | 29.85 | 30.03 | 75 | 70 | N | N | Cloud. | Fine | Cloud. |
| 19 | | | 60 | 68 | 48 | 30.10 | 30.06 | 72 | 68 | NW | NNE | Cloud. | Cloud. | Fine |

The quantity of rain fallen in the month of July, was 4.100ths of an inch.

